# Address of Prof. E. V. Stoddard, M. D., to the Graduating Class of the Buffalo Medical College, February 20th, 1877

**Published:** 1877-04

**Authors:** 


BUFFALO

Medical and Surgical Journal.

VOL XVI. APRIL, 1877. No. 9.

### Original Communications.

:o:

ART. I.—Address of Prof. E. V. Stoddard to the Graduating Class of Buffalo Medical College, Feb. 20th, 1877.

*Gentlemen of the Graduating Class:*

The Olympic festivals of Greece called together representative men of thought and power in the nation; so our college anniversaries call together those of the profession who are giving it its strength and character.

We do not meet for games of skill and strength, nor do we, like the "Knights of the Round Table," gather for a tournament and exhibition of chivalric prowess. Our anniversary is one of quiet retrospect and hopeful prospect.

We meet to-day to read one more chapter in the history of the medical scholar; to inquire what light another year, with its changing events, has shed on his character and hopes; and to impart to those who are about to go forth from the fostering care of our Alma Mater, hope, confidence and inspiration. We find that we are working together to stem the tide of ignorance which we encounter on every side, and to develop and foster the growth of the tree of science.

The college is the center of influence in the midst of this struggle. The men whom the college educates are the men who go forth to join in the strife. They have not been educated for themselves alone, but to fill a sphere in life.

It is not assumed that every graduate leaves the college, like Minerva from the brain of Jupiter, fully armed and equipped for every emergency; but that he has been so educated that he may stand in society as a source of influence, and as a center from which truth shall radiate. The scholarship which has been held up as his ideal has been that which shall expand the mind, enrich the memory, develop the intellectual power and make him the untiring investigator in the search for truth.

There can be no higher mission for true scholarship than to awaken the public conscience to the advancement of the public good. It is the scholar as the representative of thought. It is the scholar as the disciple of truth! It matters not whether this thought and truth come from the teachings of Cicero, the art of Michael Angelo, the philosophy of Aristotle, or the science of Newton, of Faraday or Tyndall. Scholarship, to be effectual, must be aggressive; it must, with a consciousness of right, nail upon the door of the temple of learning its theses, and challenge the world to judge their truth.

In addressing you on this occasion it has seemed fitting to ask your consideration of "*the claims of the profession on the medical scholar of to-day.*"

The developments of science and of every department of learning have opened a new world to the student of to-day. Before him lies an enchanted land, where mysteries hitherto forbidden fall into the hand, and heights hitherto unattainable spread out before the feet. Yet, with all this wonderful advance in expansion of our knowledge, there stand before us great questions to be answered, which our increasing knowledge shows in broader relations and more important aspects. With our growing appreciation of the character and causes of disease we are rapidly learning that, within the domain of Preventive Medicine, far more of the possible lies than in that of Curative Medicine. While we congratulate ourselves upon the progress made, we recognize the fact that our problems are but partially solved and that great questions stand before us, like the obelisks of antiquity covered with hieroglyphics, whose answer will tax our utmost effort and call into service our keenest powers of analysis.

As our civilization advances and becomes more complex in character, we note with anxiety that the physical and mental condition of man, as regards health, are depreciated. It becomes us to look well and earnestly into the causes of this condition. Thus the great questions which meet us at the outset are in the field of Sanitary or Preventive Medicine.

Hœcker has truly said, "the State which founds its legislation upon a knowledge of realities, which expects from the physical sciences information respecting human life collectively considered in all its relations, has a right to demand from its physicians a general insight into the nature and cause of popular disease."

The present year is one eminently suggestive to us. As we pause, and look backward over the foot-prints which have been left by the fleet-footed years of the century just completed, and note the development of national wealth, power and intellectual culture, we are struck with the fact that the brilliancy of our civilization is dimmed by a rapidly growing cloud. So rapidly does this cloud assume threatening proportions that it becomes us to look well toward averting the rising storm. The danger lies in the fact that, as our civilization becomes more elevated and complex in character, the physical and mental conditions of man, as regards health, are depreciated; and phases of physical and mental disease are the result.

What is there in our civilization which has this tendency, and what are the evils to be met?

Civilization we consider as simply the result of the struggle between man and the circumstances of his environment. External to man are certain physical forces constantly in operation, which tend to impair and destroy his organism. Within the man are certain vital and intellectual forces which are as constantly at work to resist the external destructive force. Between these two forces a ceaseless strife is maintained.

As man gains the ascendency, and becomes superior to the depressing circumstances of his environment, there results a condition which we call civilization; which is elevated in proportion to man's power of proving himself superior to his surroundings. Thus we have man's modifying nature, and nature modifying man, and out of this reciprocal modification what we recognize as civilization must emanate.

We must look then to the surroundings of man to find an explanation of the depressing and destructive tendencies to which I have referred.

It is the learning to know man through the medium of nature, through a thorough understanding of the circumstances of his environment, and the relation of his life to them, that must develop scientific medicine. It is in this way only that we can become truly observers of nature, thus fulfilling the province of the physician. We are daily perceiving a fuller application of the looking to the relations of man to his surroundings in the prevention of disease. The future of medicine lies open to this direction, and it is in this field that its great victories are to be gained.

We shall accomplish far more in preventing the occurrence of disease, in our communities, than in curing it in individuals. Indeed, we already see it in the decreasing mortality rates, in some of our large cities, in comparing the death rate of to-day with that of even half a century since. It may be thought that the prospect is too cheering, that I assume too much of future successes for the science of medicine. It may be urged that from the commencement of man's history he has yielded to his passions, and, as a result, has developed new forms and complications of disease, and what has been done in the past must be repeated in the future, as long as his nature remains the same. That if all disease were to be swept from the face of the earth, man would reproduce it almost immediately by his injudicious acts. Truly would such be the case, if no change were made in the training and constitution of man. But it is not too much to hope that, from the medical science of the future, there shall emanate influences which shall steadily modify the conduct of man's life, by entering that province which heretofore has been left to the philosopher and the theologian. Not that medicine is to trespass within the domain, which should be properly occupied otherwise, but by so educating man as to the bearing of his nature and relations that may he act with more wisdom. As we admit that, in nature, a process of evolution has gone on and is ever progressing, so with man, who is but a part of nature, we must admit that a similar evolution has been and is going on. We must then study man as we study nature.

We must look back over the historical evolution of the race, and search out how he has gradually grown to the capacity of the present. In such a study, we learn that the highest development of the intellect, the fullest manifestations of his faculties have been matters of growth and gradual evolution, and not the result of an implantation fully formed and expanded. That they have been slowly won as the victories of prolonged struggles and experiences. It is for us then to find out, by exact study, the laws which have been at work in the past, and by careful method apply them, with intelligent purpose, for the future higher development of the race. One of our most earnest efforts should be to impress upon our communities the power which man has over his own destiny, to pronounce his own condition as one of health or disease.

I have referred to the increasing complexity of forms of physical and mental disease, which have appeared with the development of civilization, and their threatening aspect. It is to meet this evil that I have urged the necessity for a deeper and more careful study of man's relations to his surroundings. We know that excessive use of any organ or part of the body induces special forms of disease. The same law holds in regard to the nervous system. Within certain limits, use and work develop growth and endurance; but, beyond this, injury must ensue. The capacity for intense and sustained work has not increased co-equally with the demands made for it, by the greatly developed complexity of the phases of our civilization. Look at it from what point of view we choose, the conclusion is inevitably forced upon us, that the amount and variety of nervous disease has largely increased. While physical causes of disease have been so carefully studied, that we have gained much in our power over the once destructive epidemics which swept with such fatal fury over whole nations; over the increase and development of mental disease we have achieved less power.

The causes of mental disease are less tangible and less easily recognized than those of physical disease; for them we must search through the *social* structure. As the profession of medicine becomes more scientific, and thus more thoroughly the educator of the masses, our communities will learn that all impurity, (physical, moral or intellectual,) is a deadly element; that man cannot violate physical, moral or intellectual law without injury to health or life. That ignorance or disregard of the laws governing moral and intellectual health are as certainly visited by a penalty as in the violation of the laws of physical health.

The importance of Heredity in the production of disease must not only be more deeply studied by the profession, but impressed upon the people as a part of their duty in the conduct of life. They must be taught that the mental constitutions of the parents, subject to the immense strain of modern social life, are transmitted in all their imperfections to their off-spring; and that the increase of mental disorders, of every kind, is, in a large measure, due to ignorance and a gross neglect of the laws of hereditary transmission. The increase in the forms of insanity, during the prst half century, has been largely due to the two causes: excessive mental strain and hereditary transmission. We learn thus, that the medical profession must become the guardian of the mental and intellectual, as well as of the physical, health of society. It will take the child at birth and make up his life prospects from the past and present of its family history, and its actual surroundings.

Here you will meet one of the most difficult parts of your task as public educators. You will, again and again, be disappointed and foiled by the slight interest and responsibility felt by the community in the power they possess, in controlling their physical and mental diseases. While, in the selection and propagation of their domestic animals, they exercise the greatest care, they fail to apply the same laws to their own species. While we strive to impress this all-important truth upon our people, we must, at the same time, labor to develop a greater certainty and accuracy in our knowledge of the laws of transmission, that we may be enabled to command their attention and respect, by the exactness of our knowledge, in the certainty of our predictions. It is one of the most urgent duties of medical study to develop these laws of heredity, and apply them to the improvement of the race physically, morally and intellectually.

Nor is the law of heredity of interest to the medical profession alone. The political economist realizes the importance and value of health to the State. He recognizes the extensive influence and bearing which this law exerts, in the production of pauperism and disease. He finds, among our public institutions for the care of our pauper insane, that from the effects of hereditary transmission, pauperism is handed down as a *legacy* from generation to generation. It is a startling fact to observe, that, in some of our county almshouses, two and even three generations of the same family exist as inmates, laboring under physical or mental disease directly traced to transmission.

With these patent and growing evils before us, existing in society, the responsibility of the medical profession is apparent. To search out their causes, to learn the laws of their growth, becomes the urgent duty of every student of our science, for from it must come the remedy.

These, briefly presented, are some of the questions in medical science which the future must develop and explain. And you, who are to be identified with the science of the future, cannot too earnestly turn your attention and thought in these directions But, aside from these suggestions, as to the character of your work and its province, there are other considerations, bearing upon your individual relation to your profession and to society, which will largely determine your success.

These are found in the influence which the college exerts in its training of the scholar. The forms of error, the theories of life, the problems of society, are ever changing. It is, and has ever been, the province of the University to gather and investigate the causes and tendencies of these questions in man's evolution, and to treasure up and preserve all that is of value in the recorded experiences of the past.

You have been largely under these influence, and your training has been shaped by the lines of thought and opinion which the University has established. I have said that the influence of the college is a power in society. That power has its origin in the conservatism of learning. Conservatism enters into the very idea of the university. Its learning is largely traditionary. Its inspiration is drawn from the examples of the past. The traditions of learning are the conceptions, which broad and comprehensive minds have formed, of the relations of life. The scholar is not the advocate of conservatism for its own sake; but his is conservative because his views come to him from the past. He views the relations of things in the present, through the medium of the sagacious minds of the past. Conservatism necessarily inspires caution. This is the conservatism which is the outgrowth of learning, and the power, of the scholar of to-day, is largely due to the conservative character of his study and discipline. At no time has the tendency toward liberality of thought been more marked than at the present. Old and time-honored theories have faded from view. Opinions which have been tenaciously held and accepted, with the zeal of a blind faith, have been rudely questioned and tested by rigid and searching analysis. The announcement of a fact in science, or in any department of learning, is immediately met with a searching question as to its truth. So vigorous, bold and iconoclastic has this habit of thought proved, that a broad skepticism has seemed imminent.

But, as threatening as it may have seemed, we may be content to trust to the educated men of the time to meet the evil, to sift the true from the false, and to establish truth more firmly from the very struggle necessary to maintain it. It is the scholar, with his conservative culture, who is to meet and overcome the evil. It is his culture, which has imbued him with right views of human relations, that will enable him to prove himself the safe and trusty guide. It is not alone the intellectual discipline, not alone the careful training which he may undergo, in various departments of learning, which will give him this capacity. The *whole* man must be cared for. The sentiments, the tastes, and the philosophic spirit must be cultivated. And this brings me to speak of the *character* of the culture demanded of the scholar of to-day.

Above and beyond special professional training, there is such a thing as culture and enlargement. The man must be *more* than his trade. It is necessary to every man to throw himself, with vigor, into some profession or pursuit. But there is something more ennobling, and which must ever be kept in sight, if you would prove yourselves more than useful instruments or machines. The professional man who, over and above his daily duties and business relations, has learned to feel that he has other relations, far-reaching and more permanent with his fellow-beings in all ages —that he is a debtor to a wider circle of things than the immediate circumstances of his environment, is on that account none the worse workman, and must certainly be a nobler type of man.

Culture is not the product of study alone. Learning may be acquired from books, but not so culture. It is a mere living process. It requires of the student that he close his books, go out from his closet, and mingle with his fellow men. The intercourse with living hearts must supplement his association with dead books. Man is not all intellect. He feels as well as thinks. The sublime and beautiful appeal to him as surely as the true. The moral and emotional nature cannot be separated from the intellectual. The emotions are the motive power which push the intellect into action. The ideal of culture, then, which I would have you ever keep before you, is that which comprehends the full and symmetrical development of the whole man. Your lives and character will be determined mainly by your ideal.

This ideal, whatever it may be, fusing itself into the man, acting as his source of inspiration, forms his true and essential nature and reveals itself in all his thoughts and acts. We are told, and truly, that it is not the educated alone who have their ideals—that every man, even the most ignorant, has an ideal, whether he be aware of it or not; that is, that every man has something which is the ruling thought of his life. Even the beggar in his rags has his ideal, though it be only sufficient to eat, drink and to clothe himself. Tne ideal may be material or spiritual. The kind and measure of each man's gifts with culture must determine its character. If then it be true that every man has his ideal how important is its character in determining his success. Our culture then must include with its high ideal a practical training of the hand and heart; and this training must be continuous and severe, for it is only through long and earnest discipline that the ideal may be reached, through and over the obstacles which surround its attainment.

The tendency in study and observation at the present time is largely toward *specialty*. And of the dangers here to be met I would warn you. There is a fashion in thought as well as in dress. Exclusive study, early in the life of the scholar, begets one-sidedness and flippancy. The limited knowledge, of the immature mind, begets self-sufficiency and a lessened power of broadly appreciating the relations of the subjects of observation. It is an unfailing law of our nature, which will vindicate itself, that in the exclusive devotion to one cause or one principle, the character loses its balance and steadiness, and falls, from weakness and gradual decay.

And what is true of the individual is also true of nations. The Greeks were the most imaginative of people. Taste was cultivated as the supreme guide. It was the *beautiful* at which they ever aimed. And in their history our law is verified. The exclusive devotion to one view of life led to their decay. How great the contrast formed by the Romans! Their devotion was toward organization, and *law* was their contribution to the world. Yet their exclusive devotion to one principle unbalanced by others, made them weak, and ultimately led to their decline.

Guard well then against the allurements of too early specialization in study, that the proportion and harmony of your culture may be preserved.

I have referred to the excellence of the training to which you have been subject, in the application of scientific method to observation. But here I would urge one word of caution. The various departments of science are but arbitrary divisions. Nature is not thus divided. Her complexity has compelled us to adopt such departments of observation for our convenience, not because they exist in her domain. Yet, I would urge you to follow your science from a desire to carry, into all your relations, its modes of analysis and study. Nature is one and continuous, and the modes of study in one part are applicable to others.

The fact, that science has required this, has developed a spirit very hostile to it, because, in applying to the development of man, physically and morally, the processes of investigation, applied to other parts of nature, it seemed to pull him down from the position which he had unwarrantably assumed.

We need have no fear if advancing knowledge and greater light show the error of long accepted theories. We may rest assured that the *truth* in them will live; it *must* be eternal.

And this leads me to refer to the fact, that science has given cause for offence, in demanding that the processes of observation, which it follows, should be applied to other departments of study. In a field in which metaphysical study only has been followed, physiological observations cannot be introduced, without rudely jarring some long accepted theory. If we find that nature's laws do not sustain the long venerated opinion, let us not, in a spirit of vandalism, enter upon a career of destruction; but, with the true conservation of the scholar, let the work be pursued calmly and charitably. The suspicious attitude of society, toward science, calls for nothing more than a dignified demonstration of the facts which it announces. The student of science should be content to remain within his own domain of fact and observation. It can be no satisfaction, to the earnest scholar, to hasten the decay of long venerated beliefs and doctrines. It matters not that they may fail to accord with the facts of recent observation.

They have, from time immemorial, served as an unfailing support in the moral evolution of the race, and for this reason, if for no other, should be kindly dealt with. The real object, of the scientific observer, is the elevation of mankind to a higher life. There are other laborers earnestly striving, in other fields, to attain the same end. While science strives for the elevation of the *intellect*, other influences must develop the *emotional* or *æsthetic* part. And he who strives in this other and less brilliant sphere, struggling with doubts and fears, perhaps in obscurity and privation, is none the less honorable than he who wins the plaudits of the world, by his brilliant labors and achievements, in the field of demonstrable fact. Indeed, science is independent upon these very laborers, for co-operation in its own work in elevating man. Its plain, and sometimes uninviting, truths, accepted by the philosopher and theolegian, and clothed by them with an æsthetic beauty and attraction, will often be more eagerly accepted than if presented as a mathematical proposition, appealing to the head and ignoring the heart.

In the development of the mental and intellectual, do not lose sight of the moral. The close study of science need not lead to materialism. The careful study of the development of the works of creation should not lessen, but rather strengthen, the belief in a creator. No matter how deeply we may push our studies into the evolution of nature, there is ever *something beyond*, a vast, ominscient power, in the contemplation of which man's littleness and feebleness compels him to look upward and say THERE IS A GOD!

You have chosen a profession, which, absorbing in its interest and ennobling in its study and pursuit, is not attended with great pecuniary reward. If your choice has been made from proper motives, you will have no cause for regret. It cannot be undertaken from ambitious impulses, or pecuniary considerations. Instead of proving ennobling, it must, if these considerations enter as a motive, prove demoralizing in the extreme. If the choice be made from an earnest desire to improve man's condition, and to develop truth, the fullest satisfaction and amplest scope are attained.

You are now to change your life of quiet study for one of action. Those who have followed you with interest heretofore, and sought for promise of capacity, will now look for the fulfillment of their hopes in an exhibition, in the future, of a persevering and manly use of what capacity each of you may possess.

The character of the training and study, to which you have been subject, has been such as to bring you directly into contact with the facts of nature. Nature's laws are learned by direct study and observation. Commencing with the simplest, it rises to the more complex, following thus, the law of development or evolution. You will thus keep constantly in mind the unity or continuity of nature. Man is but a part of nature, and must be studied with it. He cannot be studied apart from his relations to it.

Your advantages have exceeded those who have, even lately, preceded you. The scientific thought, the literary culture and the recorded experience of the past have been placed at your disposal. Your studies have enabled you to attain, in a day, to knowledge which has only been reached by centuries of unremitting labor and of repeated disappointment. Critical study has gained for you what has proved a painful failure, in the experience of many great minds in the past. It is for you then to show, by your future career, whether the labor spent in your training and the great advantages offered you have been in vain. I trust that you enter upon your work, with a due appreciation of your responsibility to your profession and society.

The career before you is clothed in uncertainty. For him, who shall show himself capable and trustworthy, society has a place; and success awaits. For others, who may prove unfaithful or incapable, a life of disappointment and obscurity lies before. The *law of natural selection* will be applied to you. The *survival of the fittest* will as surely prove the law for you, as it does in the organic world in the struggle for existence.

Ever aim at the practicable and possible. Do not allow an inordinate ambition to lead you beyond the limits of reason, but strive to keep within the bounds of a laudable zeal. Avoid extremes. The teaching to which you have been subject has been all in this direction. While the effort has been to encourage you to keep abreast with the spirit of the times, to investigate and observe, yet the effort of the college, in its instruction, has been to impress you with the importance of testing all facts presented with the touch-stone of truth. This is the influence which you are to carry forth with you to your labor among men. With *truth* as the test you will have an unfailing means of deciding the value of the phenomena which you may observe.

The legitimate goal of all human study is the endowment of human life with new riches, new beauties, new inventions, whether in science, religion or law. Seek then to add something to knowledge in original work. Be not satisfied with gleaning from the observations of others, but follow out lines of thought and investigation, from which you may gather some facts to add to the sum of general knowledge. Strive to be producers as well as consumers, that you may at least keep perfect the equilibrium between production and consumption.

Ever aim to profit by your own experience. The habit of recording your observations, and submitting them to the criticism and judgment of your associates, will beget habits of critical study and analysis. You will thus take care, that your mistakes prove the most valuable sources of information. They are the most expensive of teachers, but their lessons, if rightly learned, are never forgotten. Above all, seek to maintain courteous relations with your professional brethren. The ethics of the medical profession are based upon the noblest characteristics of manhood, and cannot be too carefully studied. These principles must be borne out by a strict personal morality, which the peculiar relation of the physician to the community demands. Courage of the highest type will be required of you, to practice under the severest temptations, and yet, when misjudged, to patiently wait for the verdict which certainly comes to honesty and integrity of action.

The goal, to which you look forward hopefully, is *success.* It is a word of vast signification. It is far-reaching and enters into the very essence of your life history, temporally and spiritually. Study well its relations and comprehensiveness.

One of the elsements in success is in the choice of location. It should be made with care, and when once made should be final. It is for you, then, to bend all your energies to become master of the situation. Study closely into all its characteristics and surroundings. Make a thorough sanitary survey of your territory, that you may know, as far as practicable, all the influences which may bear upon the health of the community in which you may live. Be content and willing to pay attention to little things. Never forget that the necessity for success is almost always largely in the way of patient toil and drudgery. It is not alone the great achievements which insure success, but it depends on doing *well* even the most trivial duties.

Young men are ambitious, and, at times, led to undertake too much. Ever learn to measure well your strength before assuming a burden; but having once taken it up, do not lay it down; and the habit of success will strengthen your confidence in your own powers, while it at the same time develops them.

Success, to be real, must be enduring. Notoriety is not success. The attainment of honor, wealth or power alone, is not success. It involves something more. These are but the means to achieve that fulness of life which alone constitutes success. They are to be used for the promotion of the well-being of mankind, the elevation of the race and the development in their possessor of a nobler spirit of humanity and self-sacrifice.

